# Nationwide trends of laparoscopic pyloromyotomy in patients with infantile hypertrophic pyloric stenosis in Germany: A slow path forward

**DOI:** 10.3389/fped.2023.1149355

**Published:** 2023-04-06

**Authors:** Johannes Leonhardt, Oliver Muensterer, Ahmad Alsweed, Andrea Schmedding

**Affiliations:** ^1^ Department of Pediatric Surgery and Pediatric Urology, Klinikum Braunschweig gGmbH, Braunschweig, Germany; ^2^ Department of Pediatric Surgery, Dr. von Hauner Children's Hospital, LMU Klinikum, Munich, Germany; ^3^ Department of Pediatric Surgery and Pediatric Urology, Goethe University Frankfurt, University Hospital Frankfurt, Frankfurt, Germany

**Keywords:** laparoscopic pyloromyotomy, pediatric surgery, hypertrophic, pyloric, stenosis

## Abstract

Since its introduction, laparoscopic pyloromyotomy (LP), has become increasingly popular in many countries. We have noticed an attenuated trend in Germany. The aim of this study was to analyse the distribution of open and LP in Germany. The national database of administrative claims data of the Institute for the Remuneration System in Hospitals (InEK) was analysed regarding numbers of patients with pyloromyotomy in the years 2019–2021. The German quality reports of the hospitals of 2019 and 2020 were analyzed regarding the number of procedures performed per hospital and pediatric surgical department. A total of 2050 patients underwent pyloromyotomy. The incidence of hypertrophic pylorus stenosis (HPS) was 699 and 657 patients in 2019 and 2021, respectively. Regarding age, 31.1% were admitted before 28 days of age. LP gradually increased from 216 patients (30.9%) in 2019 to 239 patients (36.4%) in 2021. Thirty-three laparoscopic operations (4.8%) were converted to an open approach. In 24 of all patients, there was an injury to the stomach, in 20 patients to the duodenum, needing repair with sutures. Analysis of the quality reports indicated that 44% of pediatric surgical departments performed LP. Although LP has became more prevalent in Germany recently, about two thirds of patients still undergo an open procedure.

## 1. Introduction

Since its description by K. Ramstedt over a century ago, pyloromyotomy has become the standard procedure for infantile hypertrophic pyloric stenosis in infants and the most common surgical procedure of the stomach in childhood (1). The incidence of this operation in Germany decreased from the maximum of 2.96 in 2006 to a minimum of 1.62 pyloromyotomies per 1,000 male live births per year in 2016 (2). Hildanus first described infantile hypertrophic pyloric stenosis in 1,646. In 1,888 Harald Hirschsprung described the clinical symptoms, the pathophysiology and conservative therapeutic approaches (1). The traditional approach for this operation is a small laparotomy. The first description of the pyloromyotomy in three newborns was published in 1908 from Fredet from France (3) before the German Surgeons Weber and Ramstedt published their small patient series 2 and 5 years later (1). The surgical technique in principal has not changed substantially over the time, but in the last 20 years other approaches, such as circumumbilical incision have been described (Bianci) (4).

Laparoscopic pyloromyotomy was described by Alain et al. in 1990 (5). Several studies describe the learning curve of the minimally invasive operation for of pyloric stenosis. The advantages of this surgical method which were seen in literature, are better cosmetics, faster recovery, faster diet, less need for analgesics and shorter hospital stay (6–8),. On the other hand, in the paper by Hall et al., in 2,830 surgeries (1,028 were open and 1,802 were minimally invasive), incomplete pyloromyotomy was found to be slightly more common with the laparoscopic method (6). The second typical complication of this operation, mucosa injury, occurred equally rare in both groups (0.29% for open surgery, 0.83% for minimally invasive surgery) (6).

Pediatric surgical care in Germany is decentralized (9). Currently, there are 90 departments and 43 smaller units distributed throughout the country (10). Furthermore, pyloromyotomy in children is mainly performed by pediatric surgeons, but also by general surgeons in some hospitals without pediatric surgery. Aim of this study was to analyze the contemporary allocation and preference of open vs. laparoscopic pyloromyotomy in Germany with the focus on complications during or after surgery and the caseload of the hospitals.

## 2. Material and methods

The study consisted of two parts: First, the analysis of surgeries performed in children under one year of age and second the analysis of the hospitals providing pyloromyotomy. Coding of the diagnoses was carried out with the International Classification of Diseases (ICD)-10 Code, German modification (11). Procedures were coded using the German procedure classification (OPS) (12). In 2019, the codes for laparoscopic pyloromyotomy were added to the OPS-catalogue, therefore data of the years 2019 and later were analysed.

### 2.1. Analysis of pyloromyotomies in children under one year of age

The “national database of administrative claims data of the Institute for the Remuneration System in Hospitals” (Institut für das Entgeldsystem im Krankenhaus: InEK) includes all diagnoses and procedures of patients admitted to a German hospital, stratified by age of the patients (13). Age is presented in distinct age groups. For our study we analyzed the age-groups “<28 days” and “28 days” – up to the 1st year of life, because the datasets are structured in this form. Results were cumulated by the database. For data protection rules, case frequencies below four were pooled. The data do not provide information on the specialty of the provider (e.g., pediatric surgeon or general surgeon) who performed the operation. This database was analysed for the years 2019 through 2021. All patients with the main diagnosis Q40.0 (hypertrophic pyloric stenosis) and procedures 5–432.00 (open pyloromyotomy), 5–432.01 (laparoscopic pyloromyotomy) and 5–432.02 (laparoscopic pyloromyotomy with conversion into open procedure) in the first year of life were extracted.

The primary goal of this part of the analysis was the distribution of laparoscopic vs. open procedures for the whole of Germany. The secondary goals were the analysis of the distribution between the two age-groups, the analysis of complications in all patients regardless of the operating method and the outcome reflected by the length of stay and the mortality.

### 2.2. Analysis of the hospitals providing pyloromyotomy

The “quality reports” of all hospitals in Germany are published annually since 2012 (14). In the year 2022 reports of data up to and including 2020 were available. These quality reports contain procedures performed for hospital admitted patients for each hospital department. Furthermore, it they indicate whether a fully trained pediatric surgeon is working at the particular hospital in question. The quality reports of the hospitals of 2019 and 2020 were analysed regarding numbers of procedures of open and laparoscopic pyloromyotomy. Data of all hospitals with a fully trained pediatric surgeon on staff were obtained. Procedures, which could be assigned to adult medicine (e.g., provided in an internal medical department), were excluded for lack of plausibility. All numbers, which were divided between the different departments, e.g., pediatric, neonatology and pediatric surgery, were summarized as one number. For data protection rules, case frequencies below four were pooled and analysed together.

The primary goal of this part of the analysis was the distribution of procedures between the centers of pediatric surgery.

### 2.3. Statistics

Statistical analysis were done with Excel ® and R, Version 4.1.2 (15). We used descriptive statistics where appropriate. Chi-squared test was used for group comparison. For the correlation between numbers of pediatric surgeons in a department to number of pyloromyotomies, we used Pearson's correlation, single-sided, *p*-values < 0.05 were considered statistically significant.

### 2.4. Ethics

An ethics vote was not required for this retrospective analysis of publicly available cumulative hospital data.

## 3. Results

### 3.1. Analysis of the national database on administrative claims data for hospital patients of Germany (INeK)

**Figure 1:**
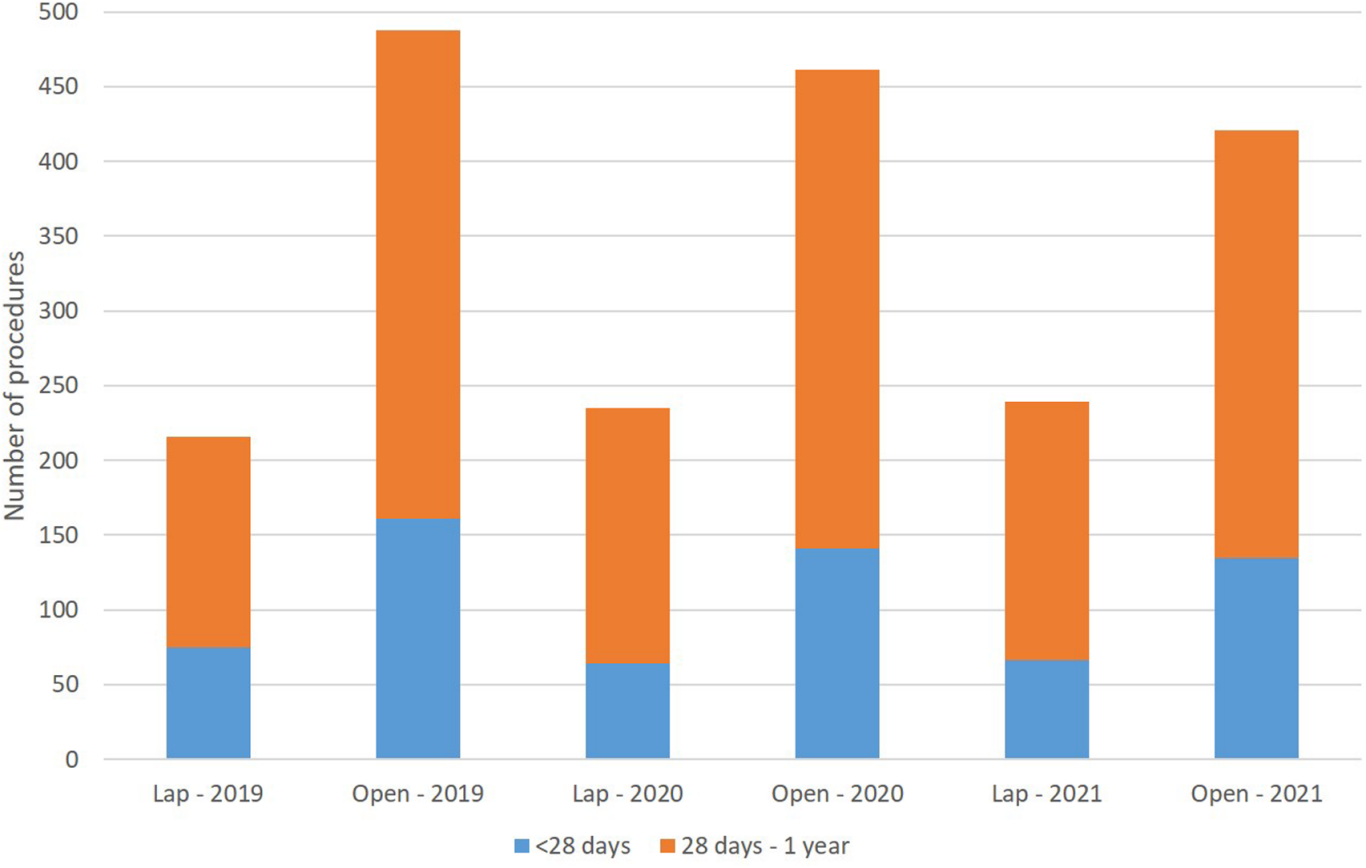
Number of patients undergoing laparoscopic or open pyloromyotomy (differences between age and surgical approach were not significant).

From January 2019 through December 2021, a total of 2050 patients under the age of one underwent pyloromyotomy due to infantile hypertrophic pyloric stenosis in Germany. The numbers declined from 699 patients in 2019 to 694 in 2020, and further to 657 patients in 2021. Of the included patients, 85.5% were male and 14.5% were female (Table 1). Regarding age, 31.1% of all patients were admitted before 28 days of age. LP was performed in 690 patients (32.3%) with an increase from 216 patients (30.9%) in 2019 to 239 patients (36.4%) in 2021 (Figure 1).

**Table 1:** Clinical characteristics of children who underwent open (5–432.00) or **lap**aroscopic (5–432.01 and 5–432.02) pyloromyotomy in Germany 2019–21. All procedures were assigned to patients not to procedures. 11 patients had both kind of procedures.

	All	%	Open	%	Lap	%
**No. of patients**						
All	2,050		1,371	67.7%	690	32.3%
Female	297	14.5%	207	15.1%	90	13.0%
Male	1753	85.5%	1164	84.9%	600	87.0%
**No. of procedures**						
5–432.00 open operation	1,381	67.4%	1,380		9	1.3%
5–432.01 laparoscopic operation	674	32.9%	8	0.6%	674	97.7%
5–432.02 conversion	33	1.6%	3	0.2%	33	4.8%
**No. of patients**						
Re-operation (5–983)	37	1.8%	22	1.6%	22	3.2%
Conversion (5–432.02)	33	1.6%			33	4.8%
Suture of stomach (5–448.00)	24	1.2%	12	0.9%	12	1.7%
Suture of duodenum (5–467.00)	20	1.0%	15	1.1%	8	1.2%
Blood transfusion (8–800.c0)	37	1.8%	25	1.8%	15	2.2%
Central venous line (8–831.0)	47	2.3%	34	2.5%	18	2.6%
Complex Intensive Care Procedure (8–98d.0)	58	2.8%	31	2.3%	29	4.2%
Length of stay (days)	6.3		6.7		5.7	

Thirty-one patients needed more than one procedure during the in-hospital stay, seven had an open pyloromyotomy twice, and five had a laparoscopic re-pyloromyotomy (Table 2). The laparoscopic approach was converted to open in 33 patients (4.8%). In 37 patients, the code for reoperation (5–983) was used. In 24 patients, there was an injury to the stomach, and 20 patients had an injury to the duodenum, needing suture repair. Transfusion of blood was given in 37 patients; 47 patients had a central venous line placed. The code for complex intensive care (8–98d.0) was employed 58 times. Mean length of stay (LOS) was 6.7 days with open surgery and 5.7 days with LP. There was no mortality in this series.

**Table 2:** Number of patients with different procedures documented, open pyloromyotomy (open, 5–432.00), laparoscopic pyloromyotomy (lap, 5–432.01) and laparoscopic pyloromyotomy with conversion into open procedure (conversion, 5–432.02).

		More than one procedures		
	One procedure	Open	Lap	Conversion
Open	1,350	7	11	3
Lap	644	–	5	5
Conversion	25	–	–	0

### 3.2. Analysis of the quality reports of Germany

In 2019, 129 hospitals had a fully trained pediatric surgeon on staff who performed pyloromyotomies decreasing to 125 hospitals in 2020. (Table 3) During the same time, there were 1,653 procedures for pyloromyotomy coded for all age groups and diagnoses in the InEK-database, of which 1,419 were for pyloromyotomy under one year of age and with the main diagnoses of hypertrophic pyloric stenosis.

**Table 3:** Number of hospitals and procedures published in the quality reports of the hospitals.

	All		Open pyloromyotomy		Laparoscopic pyloromyotomy	
Year of statistics	2019	2020	2019	2020	2019	2020
No. of hospitals	129	125	111	98	48	47
No. of procedures	576	551	375	357	201	194
No. of conversions	10	11			10 (5.0%)	11 (5.7%)
**No. of procedures per hospital**						
Median	3	3	2	2	1.5	2
Range	1–19	1–26	1–12	1–15	1–18	1–25

**Figure 2:**
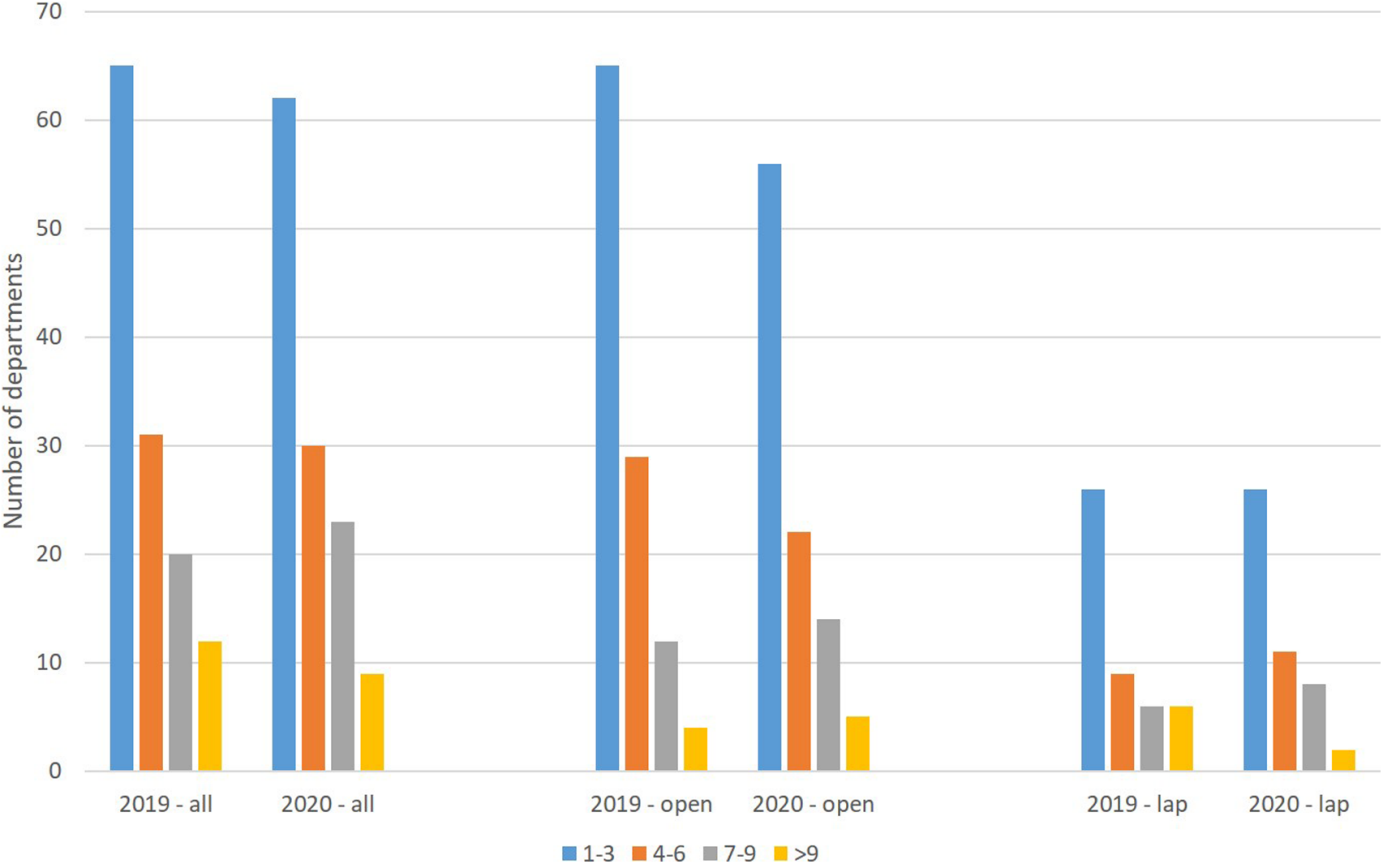
Number of procedures per hospital (*n* = 129 hospitals in 2019, *n* = 125 hospitals in 2020).

In 2019, four hospitals performed more than nine open procedures, and six performed more than nine laparoscopic procedures, in 2020 there were five vs. two hospitals, with such patient volume respectively (Figure 2).

**Figure 3:**
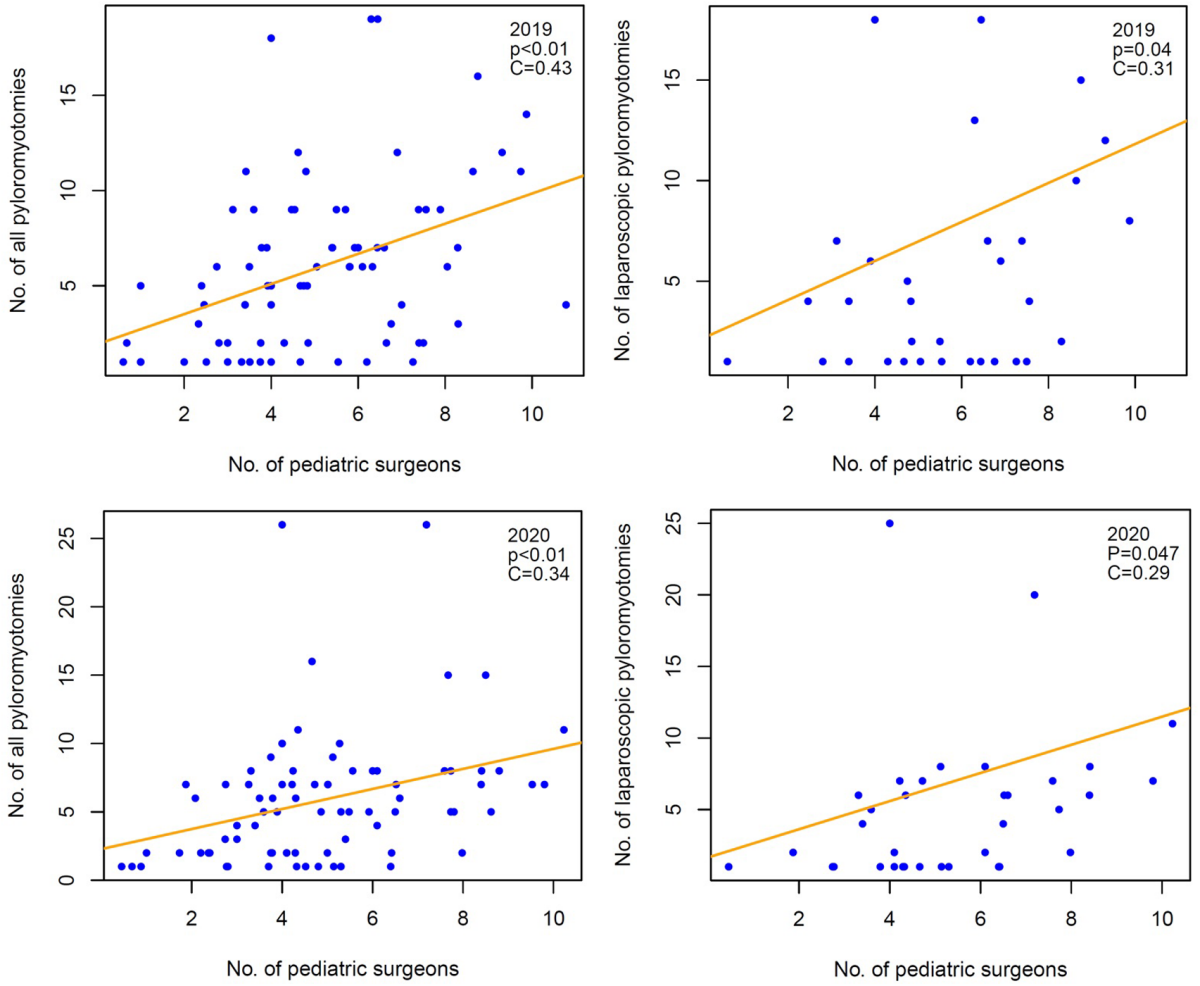
Weak correlation between number of all and laparoscopic pyloromyotomies and number of pediatric surgeons.

Pediatric surgical departments, which could be identified by their department structure in InEK, were analysed separately. In 2019 and 2020 there were 79 and 82 departments, respectively. Seventy-eight departments documented pyloromyotomies in both years. These departments performed mean of 5.9 pyloromyotomies in 2019 and 5.7 pyloromyotomies in 2020. In 2019, 32 of these hospitals performed laparoscopic pyloromyotomies, increasing to 36 in 2020 (Table 4). There was a weak positive correlation between size of the department, represented by the number of pediatric surgeons, and number of pyloromyotomies as well as number of laparoscopic pyloromyotomies (Figure 3).

**Table 4:** Pyloromyotomies in pediatric surgical departments.

Year	2019	2020
No. of departments	78	78
No. of departments with laparoscopic procedures only	11 (14.1%)	18 (23.1%)
No. of departments with open procedures only	46 (58.9%)	42 (53.8%)
No of departments with open and laparoscopic procedures	21 (26.9%)	18 (23.1%)
Number of laparoscopic procedures	165	177
Mean no. of laparoscopic pyloromyotomies per department	5.2	4.9
SD	5.1	5.1
Number of open procedures	289	272
Mean no. of open pyloromyotomies per department	5.0	4.5
SD	3.1	3.6

## 4. Discussion

30 years after the introduction of minimalinvasive pyloromyotomy, our study shows for the first time that the majority of children with infantile pyloric stenosis in Germany still undergo open surgery. Even if a trend can be appreciated towards more minimally invasive surgery in the years 2019 through2021, a laparoscopic rate of 36.4% of all operated patients is in the lower end of international reports published in the last ten years. (Table 5). Although there are some publications indicating advantages for the patients, a certain publication bias regarding this trend cannot be excluded, particularly in light of the recent Cochrane analysis (16). The lack of an overwhelming benefit may explain the slow adaption to some extent.

**Table 5:** Number of pyloromyotomies in literature compared to our data.

Year	Country	Percentage of laparoscopic procedure	No. of cases	Reference
2019–20	Germany	36.4%	2,050 (multi center)	Our data
2017–2019	Egypt	50%	80 (single center)	(6)
2013–2018	China	54%	233 (single center)	(18)
2013–2015	United States	65,5% (2015) 59% (2013)	4,847 (multi center)	(19)
2013–2014	United States	64%	3,256 (multi center)	(20)
2010–2019	Italy	43%	60 (single center)	(21)
2006–2011	England and Wales	23.7% (2011) 6.2% (2006)	9,686 (national)	(22)
2002–2013	Canada	37.2% (2013) 5,9% (2002)	4,587 (multi center)	(23)
1999–2006	United States	21.4%	622 (single center)	(24)

Similar findings have been found in a previous study on laparoscopic vs. open herniorrhaphy (17).

The rate of complications in Germany is comparable to those reported in the literature. An iatrogenic injury of the stomach or the duodenum was seen in 1.2 vs. 1.0% of all cases, comparable to the perforation rate reported of 0.48% and 3.3%, respectively (24–27). The conversion rate of 4.7% is in the range of published studies also (1). International data also confirmed minimal rates of mortality (0%–0.1%), which is again corroborated in our study (25, 27–29).

The LOS of HPS patients was longer in Germany than in most other countries. The median total LOS in our study was 6.3 days, which is in line with Cascio and Leong (30, 31) but also longer than that in other studies (25, 28, 32). In addition to the DRG system, which encourages a certain length of stay, possible reasons are the lack of possibilities to perform non-urgent surgery on a weekend. Most of the patients who are admitted on Friday are not operated on until Monday, even if the patient is ready for surgery after intravenous fluid administration. Because the insurance data used in this study do not provide length of the preoperative stay or further clinical information such as nutritional data, further research is needed to answer this question.

In 2014, Mc Ateer et al. published a study on 3,500 patients with hypertrophic pyloric stenosis, concluding that the outcome is better in hospitals with pediatric surgeons on staff than in those without. Furthermore, the volume of pyloromyotomies in a hospital with pediatric surgeons was higher than in other hospitals (33). We could not correlate outcome with the type of the department, as the InEK-Data do not report on the type of specialist performing the procedure. However, when the pediatric surgical departments were analysed separately, we found a mean number of pyloromyotomies less than six per year, which results from the high number of units performing the procedure in Germany.

As shown, pyloromyotomy is a procedure with a low rate of complications and mortality for either technique. Until now, there is no clear advantage of the minimal invasive surgery, but also no clear disadvantage (16, 34). Open pyloromyotomy can be considered the conventional standard procedure, which is usually performed by residents or even by general surgeons. As shown, the overall number of pyloromyotomies per hospital per year in Germany is quite low. This presents a challenge to build competence, experience and overcome a learning curve that generally is thought to entail at least 20 procedures. In contrast to some hospitals in US and Canada, where pediatric surgical fellows are reported to perform over 100 minimal-invasive cases during their fellowship (35), many European training centers lack this sort of patient volume (36) necessary to spread the technique throughout a department within a short time. A solution for this problem is the implementation of innovative training models using simulation and mentoring (37, 38). Centralization of the care for children with hypertrophic pyloric stenosis is currently not feasible in Germany due to the reason that it is not entirely provided only by pediatric surgeons, and the current political goal and wish of parents is to provide care close to home.

Our study has some drawbacks. Most importantly, quality reports do not distinguish between procedures for adults and minors. In the InEK-Data, 234 procedures did not meet our inclusion criteria (less than one year, and main diagnosis of HPS). Therefore, our study focused on pyloromyotomy performed in hospitals with pediatric surgeons on staff, assuming these numbers would best reflect those procedures performed in our focus group. As the InEK data only gave cumulative statistics, we could not clearly assign a complication to a procedure in cases where a patient had more than one pyloromyotomy. Another limitation of our study is the short time period over which the data was collected. The reason for this is that the billing code for laparoscopic surgery was only introduced in Germany in 2019. It is therefore not possible to collect data over a longer time period in order to be able to reliably differentiate between open and laparoscopic surgery. We acknowledge that using 3 years of data is short to describe a trend. However, it is striking that even in this short timeframe, there were clear noticeable changes in the proportion of laparoscopic pyloromyotomies performed.

## 5. Conclusion

Laparoscopic pyloromyotomy has been increasing in frequency in Germany recently, although only one third of patients undergo this minimally invasive procedure. Conversion from laparoscopic to open procedure is relatively rare and complications were similar in both groups. Reaching a plateau in the learning curve for laparoscopic pyloromyotomy is challenging in our setting, mainly due to a high number of pediatric surgeons taking care of the limited number of patients. Greater availability of training models may help in overcoming this dilemma.

## Data Availability

The raw data supporting the conclusions of this article will be made available by the authors, without undue reservation.
